# Molecular Epidemiology of Methicillin-Resistant *Staphylococcus hominis* (MRSHo): Low Clonality and Reservoirs of SCC*mec* Structural Elements

**DOI:** 10.1371/journal.pone.0021940

**Published:** 2011-07-08

**Authors:** Ons Bouchami, Assia Ben Hassen, Herminia de Lencastre, Maria Miragaia

**Affiliations:** 1 Laboratory of Molecular Genetics, Instituto de Tecnologia Química e Biológica (ITQB), Oeiras, Portugal; 2 Bone Marrow Transplant Centre of Tunisia, Tunis, Tunísia; 3 Laboratory of Microbiology, The Rockefeller University, New York, New York, United States of America; New York State Health Department and University at Albany, United States of America

## Abstract

**Background:**

Methicillin resistant *Staphylococcus hominis* (MRSHo) are important human pathogens in immunocompromised patients. However, little is known regarding its population structure and staphylococcal chromosomal cassette *mec* (SCC*mec*) content.

**Methodology/Principal Findings:**

To assess the population structure and the SCC*mec* content of *S. hominis*, 34 MRSHo and 11 methicillin-susceptible *S. hominis* (MSSHo) from neutropenic patients collected over a 3-year period were studied. The genetic backgrounds of *S. hominis* isolates were analyzed by pulsed-field gel electrophoresis (PFGE) and SCC*mec* types were determined by PCR. Cassette chromosome recombinases (*ccr*) were characterized by PCR and *ccrB* sequencing. The 34 *S. hominis* isolates were classified into as many as 28 types and 32 subtypes (SID = 99.82%); clonal dissemination was occasionally observed. The main SCC*mec* structures identified were SCC*mec* type VI (4B) (20%), SCC*mec* VIII (4A) (15%), and a new SCC*mec* composed of *mec* complex A in association with *ccrAB1* (38%); 27% of the isolates harbored non-typeable SCC*mec*. Overall, a high prevalence of *mec* complex A (73.5%), *ccrAB1* (50%) and *ccrAB4* (44%) were found. Importantly, *ccrB1* and *ccrB4* from both MRSHo and MSSHo showed a high nucleotide sequence homology with those found in *S. aureus* SCC*mec* I, VI and VIII respectively (>95%).

**Conclusions/Significance:**

The *S. hominis* population showed a limited clonality and a low genetic diversity in the allotypes of *ccr* and classes of *mec* complex. Moreover, our data suggest that *S. hominis* might have been a privileged source of *mec* complex A, *ccrB1* and *ccrB4*, for the assembly of primordial SCC*mec* types.

## Introduction

Coagulase-negative staphylococci (CoNS) are the most frequently isolated bacteria from blood cultures of febrile neutropenic patients mostly in association with the use of intravenous catheters and are a predominant cause of nosocomial infections [1]. Methicillin-resistant *S. epidermidis* (MRSE), *S. haemolyticus* (MRSHae) and *S. hominis* (MRSHo) are all capable of causing infections and usually are more likely to be multiple resistant to antimicrobial agents than other CoNS. Among staphylococci, MRSHo is of increasing concern and today represents the third most common organism among clinical isolates of MRCoNS [2], [3], [4]. In spite of its clinical significance very little information on the epidemiology of MRCoNS has been published. In particular no information is available regarding *S. hominis* population structure, genetic diversity and capacity of dissemination. The whole genome sequencing of *S. hominis* was recently completed and was determined for two strains: *S. hominis* strain SK119 and *S. hominis subsp. hominis* C80 (http://www.ncbi.nlm.nih.gov/sites/entrez?db=genomeprj&term=38759). Analysis of the nucleotide sequences available showed that both strains lack the determinant of methicillin resistance (*mecA*), but are multidrug resistant and exhibit resistance to several metal ions. Moreover, they contain a high number of transposases.

Methicillin resistance conferred by the presence of the *mecA* gene, encodes for an extra penicillin binding protein (PBP2A) with low affinity for all ß-lactams [5]. The *mecA* gene is found inside a mobile genetic element designated staphylococcal cassette chromosome *mec* (SCC*mec*) [6], composed of two essential elements; the *mec* complex that contains *mecA* and intact or truncated forms its regulators (*mecI* and *mecR1*) and the *ccr* complex composed of cassette chromosome recombinases (*ccr*) that are involved in the integration and excision of the cassette [7]. Up until now, ten major types of SCC*mec* (type I to X) have been reported in *S. aureus*, that result from specific associations between a particular *ccr* gene allotype and class of *mec* complex (A–D) [8], [9]. Several SCC-like elements that do not carry *mecA* but contain other characteristic genes (e.g., capsule gene cluster, fusidic acid resistance, or the mercury resistance operon) [8, 10, 11 12] have also been described [13], [14], [15].

Although the majority of the work on the characterization of SCC*mec* has been carried out in MRSA, this element has been described as well in other CoNS namely in *S. epidermidis*, *S. hominis*, *S. capitis*, *S. sciuri*, *S. warneri* and *S. saprophyticus* [4].

Several recent reports suggest that in CoNS, SCC*mec* structures are highly diverse. In a large study of *S. epidermidis* it was observed that SCC*mec* IV and III were the most common, however, as much as 12% of isolates carried either new or non-typeable SCC*mec* types [16]. Data on SCC*mec* carried by *S. hominis* is scarce. In the few isolates characterized so far, SCC*mec* types Ib, III and new SCC*mec* types (1A, 1+4A, 5B) have been reported [7], [17], [18], [19] as well as non-typeable SCC*mec* structures [17], [4].

Besides carrying SCC*mec* and SCC-like cassettes, CoNS were suggested to be active players in the assembly of these mobile genetic elements. Previous studies have shown that a *mecA* homologue, ubiquitous in *Staphylococcus sciuri,* may have been the evolutionary precursor of *mecA* - the structural gene encoding PBP2a [20] and that the *ccr* and *mec* complexes from an unknown source were probably brought together in other CoNS [21], before the cassette was transferred into *S. aureus* [22]. Also, the recent finding of a 99% of nucleotide sequence homology between the SCC*mec* IV from *S. epidermidis* and *S. aureus* showed that SCC*mec* IV in *S. aureus* was probably originated in *S. epidermidis* [23]. Similarly, the discovery of regions of high homology between a SCC non-*mec* containing *ccrAB1* from *S. hominis* and SCC*mec* type I from *S. aureus*, lead to the suggestion that this SCC non-*mec* from *S. hominis* could have been the primordial form of SCC*mec* I [12]. Still several links are missing in the evolutionary history of SCC*mec* and the contribution of each CoNS species to SCC*mec* evolution is not known.

In this study, we provide what we believe are the first insights into the molecular epidemiology of *S. hominis* through the description of the diversity of clonal types in MRSHo and MSSHo and SCC elements in isolates from neutropenic patients collected at the bone marrow transplant centre of Tunisia. We also provide a detailed analysis on the most likely contribution of *S. hominis* to SCC*mec* evolution.

## Materials and Methods

### Ethics Statement

This study was performed with approval from the Local Medical Ethical Committee of Charles Nicolle Hospital, Tunis, Tunisia. Since the strains were de-identified and analyzed anonymously and the strains, not a human, were studied, this is exempt from human research committee approval according to the regulations of the Local Medical Ethical Committee of Charles Nicolle Hospital, Tunis, Tunisia and informed consent is not required according to the Ethical Committee.

### Bacterial isolates

A total of 34 nosocomial MRSHo isolates consecutively sampled between 2002 and 2004 from neutropenic patients hospitalized at the bone marrow transplant centre of Tunisia, and recovered from blood cultures (29.4%), intravenous catheters (29.4%) and other specimens (41.2%), were analyzed. Isolates were hospital-acquired (specimens collected from patients hospitalized in the graft or hematological units (20), more than 48 h after admission, or from patients that had a history of hospitalization within 6 months prior the isolation date). The study included only one clinical isolate per patient. A group of 11 MSSHo collected over the same time period was included for molecular characterization of *ccr* genes.

### Control isolates

*S. aureus* ATCC25923 [24] and *S. epidermidis* RP62A [25] were included for quality control of antimicrobial susceptibility patterns. The type strain of *S. hominis* ATCC27844^T^ was used as reference for ITS-PCR identification. *S. aureus* NCTC10442, N315, 85/2082, JCSC4744, WIS and HDE288 [6], [26]–[29] were included as controls for SCC*mec* type I, II, III, IV, V and VI, respectively. *S. aureus* strain COL [30] was used as a source for the *mecA* probe and as positive control for *ccrAB1* in hybridization assays; *S. aureus* WIS [29] was additionally used as a source for the *ccrC* probe; *S. epidermidis* RP62A was used as an internal control of PFGE and as a source of *ccrAB2* probe; *S. aureus* 85/2082 was used as a positive control for *ccrAB3* and *ccrC* in hybridization assays; *S. epidermidis* ATCC12228 [31] was used as positive control for *ccrAB2* and *ccrAB4*; and HDE288 [6] was used as a source of *ccrAB4* probe.

### Species identification

All isolates were tested by conventional identification phenotypic methods: mannitol fermentation, Gram staining, catalase, coagulase tests (BBL Coagulase Plasma Rabbit test, Becton Dickinson Microbiology systems, Cockeysville, USA) and DNAase activity. Isolates were characterized at the species level by API ID 32 STAPH system (BioMérieux, Marcy l′Etoile, France) according to manufacturer's instructions. Species identification was confirmed by ITS-PCR, as described [32].

### Antimicrobial susceptibility testing

Antimicrobial susceptibility testing was performed using the disk diffusion method on Muller-Hinton agar (Difco, Detroit, USA) according to the recommendation of the French Society of Microbiology «Comité de l′Antibiogramme de la Société Française de Microbiologie» (CA-SFM) (http://www.sfm.asso.fr). Antimicrobial agents tested included penicillin G (6 µg, 10UI), oxacillin (5 µg), cefoxitin (30 µg), cotrimoxazole (1,25/23,75 µg), streptomycin (10UI), amoxicillin (25 µg), amoxicillin-clavulanic-acid (20/10 µg), gentamicin (15 µg), kanamycin (30 µg), tobramycin (10 µg), erythromycin (15UI), pristinamycin (15 µg), lincomycin (15 µg), tetracycline (30 µg), chloramphenicol (30 µg), rifampicin (30 µg), ofloxacin (5 µg), ciprofloxacin (5 µg), vancomycin (30 µg), teicoplanin (30 µg), fosfomycin (50 µg), and fusidic acid (10 µg) (Sanofi Diagnostics Pasteur).

Methicillin resistance was confirmed by oxacillin (5 µg) and cefoxitin (30 µg) disc diffusion tests after 24 h incubation at 37°C. The minimum inhibitory concentration (MIC) for oxacillin was determined by E-test (AB-biodisk, Dalvogen, Sweden) on Muller-Hinton agar (Difco, Detroit, USA) and interpreted as recommended by CA-SFM (http://www.sfm.asso.fr). Multiresistance was defined as resistance to three or more antimicrobial classes.

### DNA preparation

DNA for PFGE was prepared as described [33], [34]. Genomic DNA for PCR was extracted as described before [32]. DNA probes for *mecA*, *ccrAB1*, *ccrAB2*, *ccrAB4* and *ccrC* were prepared using previously described primers [35], [36] followed by purification by the Wizard PCR preps DNA Purification System (Promega, Madison, WI).

### Detection of the *mecA* gene

The presence of *mecA* was determined by amplification by PCR [36] and confirmed for all isolates by hybridizing the XhoI (or ClaI) restriction band patterns with a DNA probe for *mecA*.

### PFGE typing

PFGE was performed as described [33], [37] with the following modifications. XhoI (20 units/disk) was chosen as the restriction enzyme for PFGE. The running conditions were the following: block1- pulse times 2 to 20 s, running time 11 h and block2- pulse times 2 to 7 s, running time 15 h; voltage 6V; angle 120° [38]. Low Range Lambda ladder DNA (New England BioLabs, Beverly, USA) was used as molecular weight PFGE marker. *S. epidermidis* RP62A was used to access inter-gel reproducibility. XhoI PFGE restriction band patterns were analyzed by visual inspection by counting the number of band differences; and automatically using BioNumerics Software (version 4. 5) from Applied Maths (Sint-Martens-Latem, Belgium). Clusters (PFGE types) were defined using the Dice similarity coefficient and the unweighted pair group method with arithmetic means (UPGMA), with 1% of tolerance and 0.8% optimization, using a cutoff similarity value of 90%. PFGE types were identified by letters; and subtypes were identified by letters followed by a numeric subscript.

### Southern blotting and DNA hybridization

XhoI DNA fragments in PFGE gels, were transferred by vacuum blotting as previously described [39] and hybridized with a DNA probe for *mecA* using ECL direct Prime Labeling and detection systems (Amersham Biosciences, Buckinghamshire, United Kingdom), according to manufacturer's instructions.

### Analysis of SCC*mec* structure

The structures of *ccr* and *mec* complex were determined by conventional PCR reactions as described by Okuma et al. [35]. The *ccrAB4* was detected by PCR amplification using the primers described by Oliveira et al. [40]. The *mec* complex C1 was amplified by PCR using the primers defined by Katayama et al. [41]. As a first approach, SCC*mec* types in *S. hominis* were defined by the combination of the type of *ccr* complex and the class of *mec* complex [29], [35] using the guidelines proposed for *S. aureus* by the International Working Group on the Classification of Staphylococcal Cassette Chromosome Elements (IWG-SCC) [8]. SCC*mec* was considered as non-typeable when *ccr*, the *mec* complex or both were non-typeable. The *mec* complex and *ccr* complex were considered non-typeable when no PCR amplification occurred for any of the primer pairs used.

### *ccrB* typing

The *ccrB* typing was performed as previously described [42]. The *ccrB* nucleotide sequences were compared by the construction of an unrooted phylogenetic tree using the average distance clustering method and the default parameters set in the *ccrB* typing tool (http://www.ccrbtyping.net). The measurement of statistical confidence of the clustering was performed by bootstrap resampling (1,000).

### Genotypic diversity

Genotypic diversity was calculated by using Simpson's index of diversity (SID) [43].

## Results

### Optimization of PFGE typing method for *S. hominis*

**Figure 1:**
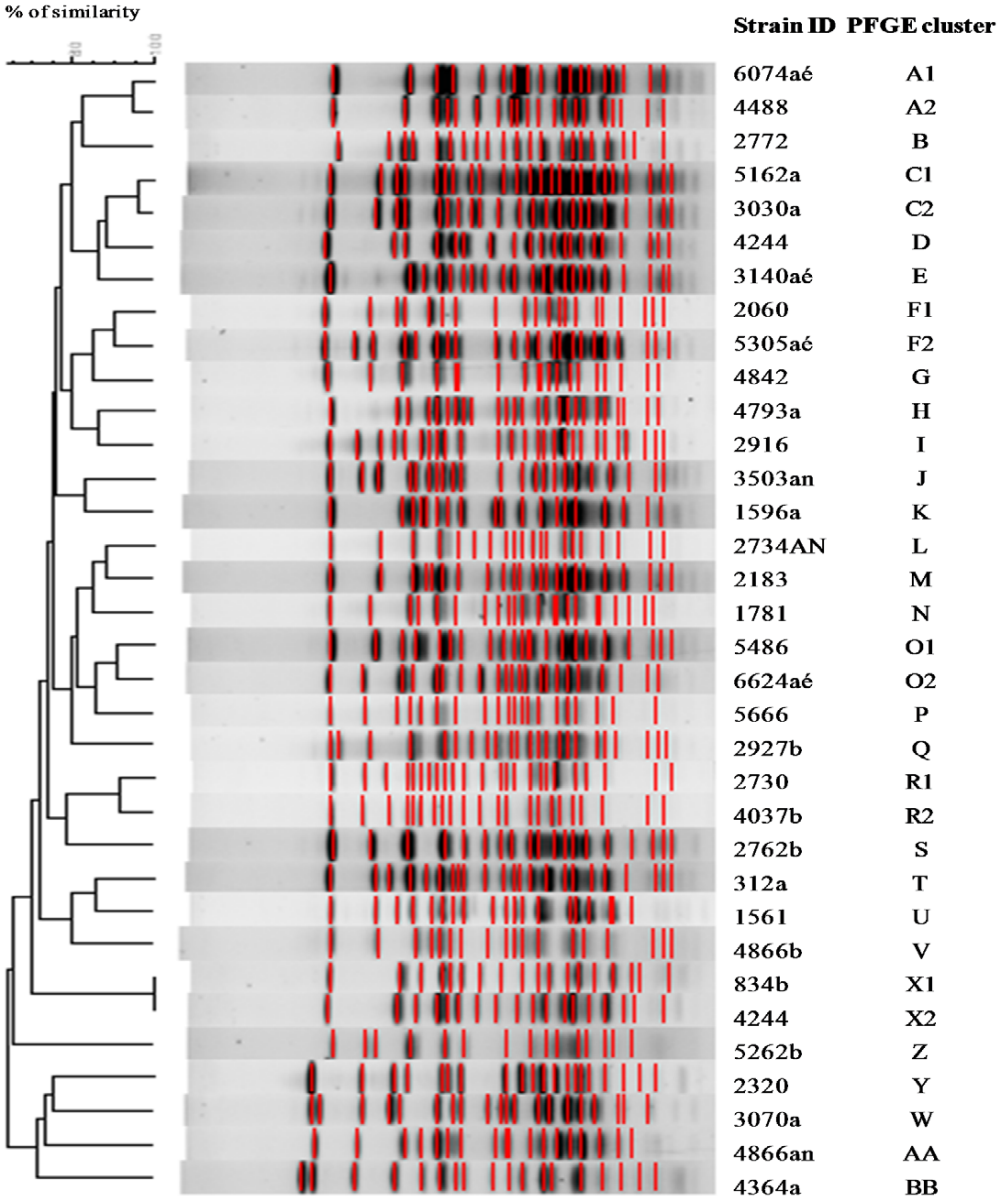
PFGE profiles after XhoI digestion of methicillin-resistant *S. hominis* isolates. The dendrogram showing the clustering of strains was performed by analysis of PFGE profiles with the Bionumerics software, with 1% tolerance and 0.8% optimization (this study). Clusters were defined at a cut-off value of 90%.

The enzyme SmaI generates a pattern of 4–5 fragments only, using the protocol defined for *S. aureus* [33], [37]. For this reason we chose the enzyme XhoI and changed the running conditions. We obtained clear, reproducible and well separated banding profiles, containing 18–20 fragments ranging from approximately 6 to 165 Kb in size (Figure 1). The parameters that provided the best concordance between visual and automatic clustering classifications were 1% of tolerance and 0.8% optimization, using a cutoff similarity value of 90% that corresponded to up to 5 bands difference in macrorestriction band patterns, when analyzed visually. Isolates were considered to belong to the same PFGE type when they were put together in the same cluster, after automatic clustering analysis, using the parameters mentioned above. Unique band patterns within each PFGE type were considered as PFGE subtypes.

### Genetic diversity and clonality of MRSHo

The molecular characterization of MRSHo isolates by PFGE classified the 34 isolates into 28 PFGE types (A–AA), revealing an extremely high genetic diversity (SID = 98.75% for the PFGE type and SID = 99.82% for the PFGE subtype) (Figure 1). Six PFGE types (A, C, F, O, R, and X) contained two isolates each, and the remaining 22 isolates were included in unique types (Table 1). The combination of clinical and typing data revealed that MRSHo belonging to the same PFGE type were found with a 3-year interval in the same unit. This is the case, for example, of isolates of PFGE types A1 and A2 that were collected from two different patients in the hematological unit in 2002 and 2004, respectively. Moreover, cross dissemination of MRSHo among patients in the same ward and across different wards was also observed. This was illustrated clearly, by isolates of PFGE type R1 and R2 that were isolated from two patients both in the Graft Unit within a three year period; and by isolates of PFGE types X that were isolated within a five month interval from one patient in the Graft Unit and another in the Hematological Unit (Table 1).

**Table 1:** Molecular and phenotypic characterization of methicillin-resistant *S. hominis* (MRSHo) isolates.

Strain	Date of	Ward^a^	Clinical^b^	MIC Ox	*mecA* c	SCC*mec* typing^d^			Resistance profile^e^	PFGE cluster
	isolation		product	(µg/ml)		*mec* complex	*ccr* type	SCC*mec*		
6074aé	31-12-02	HU	Bl	0.5	+	class B	type 4	VI	P, Ox, E, SXT, Fu	A1
4488	09-08-04	HU	Ca	0.5	+	class B	type4	VI	P, S, K, E, Rf, SXT, Fu	A2
2772	18-05-04	HU	Ca	0.38	+	class B	type 4	VI	P, Ox, Of, Cp, fo, Fu	B
5162a	09-11-02	HU	ISCa	1.5	+	class A	type 1	new1	P, Ox, Amx, G, K, T, L, Tc, SXT, Of, Cp, Fu	C1
3030a	08-07-03	HU	Ca	3	+	class A	type 1+4	NT1	P, Ox, Amx, G, K, T, L, Tc, SXT, Of, Cp, Fu	C2
4244	24-09-03	HU	Ca	0.19	+	NT	NT	NT	P, K, E, Tc	D
3140aé	18-07-03	HU	Bl	8	+	class A	type 1	new1	P, Ox, Amx, Amc, S, G, K, T,E, L, Tc, Ch, SXT, Fu	E
2060	07-05-03	GU	Ca	>256	+	class A	NT	NT	P, Ox, Amx, Amc, G, K, T, E, L, Tc, SXT, Fo, Fu	F1
5305aé	02-10-04	HU	Bl	>256	+	class A	type 1	new1	P, Ox, Amx, G, K, T, E, SXT, Of, Cp, Fu	F2
4842	24-10-02	GU	ISCa	>256	+	class A	type 4	VIII	P, Ox, Amx, G, K, T, E, L, Tc, SXT, Fo, Fu	G
4793a	21-10-02	GU	Bl	0.047	+	class A	type 4+ccrC	NT4	L	H
2916	30-06-03	GU	Ca	1.5	+	class A	type 4	VIII	P, Ox, S, G, K, T, E, Ch, SXT, Fu	I
3503an	21-06-04	GU	Bl	>256	+	class A	type 1	new1	P, Ox, G, K, T, Amc, E, SXT, Of, Cp,	J
1596a	10-04-03	HU	Ca	0.75	+	class B	type 4	VI	P, Ox, K, E, SXT, Fu	K
2734an	17-05-04	GU	Bl	4	+	class A	type 1	new1	P, Ox, T, E, L, SXT, Of, Cp, Fu	L
2183	13-05-03	HU	Ca	0.19	+	class A	type 1	new1	P, Ox, G, K, T, E, SXT, Of, Cp, fo, Fu	M
1781	22-04-03	GU	Ca	0.064	+	class A	type 1+ccrC	NT2	P, G, K, T, E, SXT, Of, Cp, Fu	N
5486	26-11-02	HU	ISCa	6	+	class A	type 1	new1	P, Ox, Amx, G, K, T, E, Tc, SXT, Fu	O1
6624aé	06-12-04	HU	Bl	6	+	class A	type 1	new1	P, Ox, G, K, T, E, L, Tc, SXT, Of, Cp, Fu	O2
5666	09-12-02	GU	ISCa	4	+	class A	type 1+ccrC	NT2	P, Ox, T, E, SXT, L, Rf, SXT, Of, Cp, Fu	P
2927b	01-07-03	GU	Ca	>256	+	class A	type 1	new1	P, Ox, Amx, Amc, G, K, T, L, SXT, Of, Cp, Fu	Q
2730	27-06-02	GU	ISCa	0.19	+	class A	type4	VIII	P, Ox, Amx, G, K, T, L, Tc, SXT, Fu	R1
4037b	20-09-02	GU	ISCa	4	+	class A	type4	VIII	P, Ox, T, E, L, SXT, Of, Cp, Fu	R2
2762b	26-06-02	HU	ISCa	24	+	class A	type4	VIII	P, Ox, Amx, G, K, T, L, Tc, SXT, Fu	S
312a	20-01-03	HU	Ca	12	+	class A	type 1	new1	P, Ox, Amx, G, K, Tc, SXT, Fu	T
1561	19-03-04	HU	Bl	4	+	class A	type 1	new1	P, Ox, G, K, T, E, Tc, SXT, Of, Cp, Fu	U
4866b	21-10-03	HU	Pu	1.5	+	class A	type 1+4	NT1	P, Ox, G, K, T, E, L, Tc, SXT, Of, Cp, fo, Fu	V
834b	11-02-04	GU	Ca	4	+	class A	type 1	new1	P, Ox, Amx, K, Tc, Rf, SXT	X1
4244	27-07-04	HU	Ca	0.094	+	NT	NT	NT	-	X2
5262b	13-11-02	HU	ISCa	>256	+	class A	NT	NT	P, Ox, Amx, G, K, T, E, Tc, SXT, Fo, Fu	Z
2320	26-04-04	HU	Bl	0.25	+	class B	type4	VI	P, Ox, Amx, Amc, K, E, L, Rf, SXT, Of, Cp, Fu	Y
3070a	31-05-04	HD	Pu	0.38	+	class B	type4	VI	P, Ox, E, Tc, SXT, Of, Cp, Fu	W
4866an	04-09-04	HU	Bl	8	+	class A	type 1	new1	P, Ox, Amx, K, T, Tc, Fu	AA

a HU: hematological unit; HG: graft unit; HD: hospital day;

b Bl: blood; Ca: catheter; ISCa: insertion site of catheter; Pu: pus;

c +, *mecA* gene present;

d NT: not typeable;

e P: penicillin G; Ox: oxacillin; S: streptomycin; G: gentamicin; K: kanamycin; T: tobramycin; E: erythromycin; L: lincomycin; Amx: amoxicillin; Amc: amoxicillin-acid clavulanic; Tc: tetracycline; Ch: chloramphenicol; Cp: ciprofloxacin; Of: ofloxacin; SXT: cotrimoxazole; Rf: rifampin; Fo: fosfomycin; Fu: fusidic acid.

In spite of the few examples of dissemination of *S. hominis* isolates, we could observe that, with the exception of two isolates (PFGE G), a unique PFGE subtype was associated to each patient. The results illustrate well the low clonality of the species.

### Antibiotic susceptibility patterns

The antibiotic susceptibility to a panel of 22 antibiotics and the MIC for oxacillin was determined for the 34 *mecA*-positive MRSHo. A total of 28 out of 34 isolates (80%) expressed phenotypic methicillin resistance (1.5 µg/ml). However, six isolates were susceptible to oxacillin in both disc diffusion and E-test (MICs 0.047–1.5 µg/ml), in spite of the fact they carried *mecA*; this discrepancy was not investigated further, but might be due to the presence of point mutations in *mecA* that can lead to the production of a less active PBP2a or two the existence of extremely heterogeneous oxacillin resistance profiles, that could not be detected by the methods used.

All MRSHo isolates were resistant to at least one of the non-β-lactam antibiotics tested, as follows: 29 (85%) were resistant to cotrimoxazole, 29 (85%) to fusidic acid, 25 (73%) to kanamycin, 23 (67%) to erythromycin, 21 (62%) to tobramycin, 18 (53%) to gentamicin, 18 (53%) to ofloxacin, 17 (50%) to ciprofloxacin, 16 (47%) to tetracycline, 15 (44%) to clindamycin, 6 (17%) to fosfomycin, 4 (12%) to rifampin and 3 (9%) to chloramphenicol. All the isolates were susceptible to pristinamycin, vancomycin and teicoplanin. As many as 94% of MRSHo isolates were multidrug-resistant.

No correlation was found between the PFGE type/subtype and the antibiogram profile. Although we found isolates with the same PFGE type (PFGE C) and the same highly resistant antibiotype, we also found cases where two isolates with exactly the same PFGE subtype (PFGE X), exhibited either resistance to seven different antimicrobial agents or susceptibility to all the antimicrobial agents tested (Table 1).

### SCC*mec* distribution in *S. hominis*

The molecular characterization of the SCC*mec* carried by MRSHo showed that SCC*mec* VI (4B) was carried by 20% of the isolates, and SCC*mec* type VIII (4A) by 15%. A high proportion of MRSHo strains (38%, 13 out of 34) carried a unique new association between the *mec* complex class and the *ccr* complex, corresponding to *ccrAB1* associated to *mec* complex type A (1A). In addition, a high number of non-typeable SCC*mec* structures (27%) were also found, that resulted from the finding of a single *mec* complex and two different *ccr* complex allotypes in the same isolate. They included: *mec* complex class A, *ccrAB1* and *ccrAB4* (NT1) (two isolates); *mec* complex class A, *ccrAB1* and *ccrC* (NT2) (two isolates); and class A, *ccrAB4* and *ccrC* (NT3) (one isolate). These results may correspond to the existence of a SCC*mec* type with two *ccr* complexes (like SCC*mec* type III), or the existence of a SCC*mec* in tandem with a SCC non-*mec*. In addition, two isolates carried *mec* complex type A but none of the *ccr* genes described so far and in two isolates neither *ccr* nor *mec* complexes were typeable, although *mecA* was present. This may correspond either to new SCC*mec* type structures or to remnants of SCC*mec* that have lost the *ccr* complex.

Overall a high frequency of *mec* complex class A (73.5%) and *ccrAB1* (50%) and *ccrAB4* (44%) was observed among MRSHo.

Regarding the distribution of SCC*mec* among the different PFGE types, we found cases where isolates with the same PFGE type (PFGE types A, O and R) carried the same SCC*mec* type (4B, 1A, and 4A, respectively). However, we also observed that the same SCC*mec* could be carried by isolates of different PFGE types. For example, SCC*mec* 1A was carried by as many as 12 different PFGE types and SCC*mec* type 4A was carried by four different PFGE types (Table 1), suggesting either multiple acquisitions of SCC*mec* or the existence of a highly adaptive and diverse clone.

### Identification of *ccrAB* and *ccrC* genes in *mecA*-negative *S. hominis* isolates

The presence of *ccr* genes among MSSHo isolates was tested by Southern hybridization followed by *ccr* typing. Among the 11 *mecA*-negative isolates analyzed, three harbored *ccrC*, two carried *ccrAB1*, two *ccrAB4,* and one isolate carried *ccrAB2*. We also found one isolate that showed to carry the *ccr* genes by Southern hybridization but that was non-typeable by *ccr* typing by PCR. All these *ccr* genes found among MSSHo probably belong to SCC non-*mec* elements. Interestingly, all except three of the isolates carrying *ccr* genes showed resistance to three or more antimicrobial classes (Table 2). From the two MSSHo isolates recently sequenced, only one of the isolates (K119) carried a cassette chromosome recombinase with high homology with *ccrC* from the SCC*mec* V, which appears to be located far away from the *orfX* region (draft sequence data). Similarly to our findings, both strains (K119 and C80) were also multidrug resistant, although they did not carry the gene *mecA*.

**Table 2:** Molecular and phenotypic characterization of the 11 methicillin-susceptible *S. hominis* (MSSHo) isolates.

Strain	Date of	Ward^a^	Clinical	MIC Ox	*mecA* c	SCCmec typing^d^			Resistance profile^e^
	isolation		Product^b^	(µg/ml)		*mec* complex	*ccr* type	SCC*mec*	
2573b	17-06-02	HU	Ca	0.19	-	NT	type4	NT	P, E, Tc
2189	13-05-03	HU	Bl	0.125	-	NT	type 1	NT	P, Ox, Amx, S, G, K, T, E, Ch, Rf, SXT, Of, Cp, Fu
3606a	21-08-03	HU	Th	0.125	-	NT	NT	NT	S
2910	30-06-03	GU	Bl	0.19	-	NT	type4	NT	P, Amx, SXT, Fu
1342b	25-03-03	GU	Ca	0.19	-	NT	type 2	NT	P, Ox, Amx, G, K, T, E, SXT, Of, Cp, Fu
1756′	21-04-03	DH	Bl	0.19	-	NT	*ccrC*	NT	P, Ox, E, L,
1839a	25-04-03	HU	Bl	0.25	-	NT	NT	NT	P, S, K, E, L, Tc,
2347a	27-04-04	HU	Ca	0.064	-	NT	*ccrC*	NT	P
2654A	13-05-04	GU	Bl	0.064	-	NT	*ccrC*	NT	-
5425	09-10-04	HU	Al	0.047	-	NT	type 1	NT	-
3707b	01-07-04	HU	Bn	0.064	-	NT	NT	NT	P, Tc, Fo

a HU: hematological unit; GU: graft unit; DH: day hospital;

b Bl: blood; Ca: catheter; Th: throat; Al: alimentation parenteral; Bn: bone fragment;

c - *mecA* gene absent;

d NT: not typeable;

e P: penicillin G; Ox: oxacillin; S: streptomycin; G: gentamicin; K: kanamycin; T: tobramycin; E: erythromycin; L: lincomycin; Amx: amoxicillin; Tc: tetracycline; Ch: chloramphenicol; Cp: ciprofloxacin; Of: ofloxacin; SXT: cotrimoxazol; Rf: rifampin; Fu: fusidic acid.

### Nucleotide homology between *ccrB* from *S. hominis* and other *Staphylococcus* species

**Figure 2:**
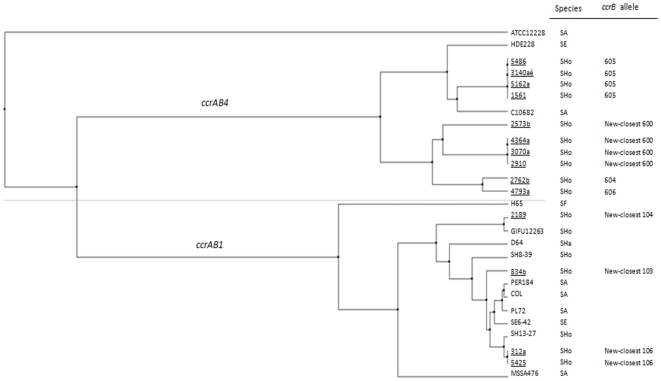
Phylogram (average distance) using the identity percentage for *ccrB*1 and *ccrB*4 carried by *S. hominis* isolates and *ccrB* prototype sequences available on 17 June 2010 at the ‘*ccrB* typing tool’ online resource. The tree was automatically drawn by the Java applet available through the ‘*ccrB* typing tool’ using the default parameters [14]. MRSHo and MSSHo isolates from this study are underlined. Control isolates were the following by order of appearance from top to bottom: ATCC12228, Methicillin-susceptible *S. epidermidis* (MSSE) strain; carrying the SCC*_pbp4_*; HDE288, methicillin-resistant *S. aureus* (MRSA), carrying SCC*mec* VI; C10682, MRSA carrying SCC*mec* type VIII; H65, *S. fleuretti* isolate from database carrying *ccrAB4* from *ccrB* typing tool database; GIFU1223, MSSHo strain GIFU1223 (ATCC27844), carrying the SCC*_1223_*; SH-8-39, *S. hominis* isolate carrying *ccrAB1* from the database; D64, *S. haemolyticus* isolate carrying *ccrAB1* from the database; PER184, MRSA, carrying SCC*mec* I; COL, MRSA carrying SCC*mec* I; PL72, MRSA carrying SCC*mec* I; SE6-42, *S. epidermidis* isolate from the database, carrying *ccrAB1*; SH13-27, *S. hominis* from the database carrying *ccrAB1*; MSSA476, methicillin-susceptible *S. aureus* (MSSA), carrying SCC*_MSSA476_*. SA, *S. aureus*; SE, *S. epidermidis*; SHo, *S. hominis*; SHa, *S. haemolyticus*; SF, *S. fleuretti*.

In order to verify if *ccrAB4* and *ccrAB1* found in *S. hominis* were similar to those previously described, *ccrB* typing was performed for all MRSHo and MSSHo carrying a single *ccrAB* allotype (28 MRSHo and 5 MSSHo). A high homology (97–99%) was found between *ccrB1* from *S. hominis* isolates from our study and those of *ccrB1* of other *S. hominis* isolates in the database (SH13-27 and SH8-39). More interesting, a similar homology was found between *ccrB1* from our collection and the *ccrB1* from *S. epidermidis* (SE6-42). Likewise, *S. hominis ccrB4* showed a high nucleotide sequence homology (92 to 96.5%) with *ccrB4* from *S. aureus* (HDE288) (Figure 2).

Thirteen out of the 33 isolates were non-typeable by *ccrB* typing methodology, because of the presence of several superimposed peaks in sequence's traces. As *ccrB* typing makes use of degenerated primers, the existence of superimposed peaks may indicate the presence of multiple *ccr* alleles of the same allotype, which the PCR multiplex methodology is unable to detect.

We also found four isolates for which PCR-based typing indicated the presence of *ccrAB1*, but *ccrB* sequencing indicated the presence of *ccrAB4* (5162a, 3140aé, 5486, and 1561). The results suggest that these isolates carry the two *ccrB* allotypes (*ccrB1* and *ccrB4*). However they could not be both detected by the same methodology most probably due to nucleotide differences in the primer regions used. Altogether, these apparently discrepant results, suggests a large pool of *ccrAB1* and *ccrAB4* alleles in *S. hominis*.

A total of four different *ccrB1* and 10 different *ccrB4* alleles were found in the 14 isolates from our collection, for which a *ccrAB* allotype could be determined. The phylogenetic relationships among *ccrB1* and *ccrB4* alleles found in this study with those of control strains available in the database [44] are displayed in Figure 2. The *ccrB4* alleles were clustered into three different branches: one containing the *ccrB4* from the SCC*pbp4* of *S. epidermidis* strain ATCC12228; the other containing SCC*mec* VI prototype *S. aureus* strain (HDE288), SCC*mec* VIII prototype *S. aureus* strain (10682) and four isolates from our study (5162a, 3140aé, 5486, and 1561); and the last one containing only four MRSHo (4793a, 2762b, 3070a, 4364a) and two MSSHo isolates (2573b, 2910) from our collection.

The *ccrB1* allele was present in several species in the *ccrB* database, being distributed in one major cluster that contained three prototype *S. aureus* strains for *ccrAB1* (COL, PER184, PL72), one *S. epidermidis* isolate (SE 6–42), one *S. hominis* (SH 13–27) isolate from the database and three *S. hominis* isolates from our collection (two MRSHo and one MSSHo). The remaining five isolates were distributed by five different branches (*S. fleuretti* H65; *S. aureus* MSSA4476, *S. hominis* 2189 from our study, *S. hominis* SH8-39, and *S. haemolyticus* D64).

The phylogenetic analysis showed that *ccrAB1* and *ccrAB4* from *S. hominis* and those found in *S. aureus* SCC*mec* are highly similar and must have had a common origin.

## Discussion

This study is the first to describe the molecular epidemiology of nosocomial *Staphylococcus hominis* species. Our data showed that *S. hominis* population was composed of a very high number of PFGE types and subtypes, suggesting that each *S. hominis* clone as defined by PFGE can eventually be host specific and is only rarely disseminated. However, we also found *S. hominis* isolates that were isolated with a 3-year interval in the same hospital. The results indicate that the great majority of *S. hominis* infections most probably have originated from the endogenous flora of patients, rather than acquired from the environment or from cross transmission. However, only the study of isolates collected from healthy persons in the community with no recent hospital contact will enable to confirm this hypothesis.

The PFGE protocol optimized in this study illustrated well the genetic diversity of *S. hominis* population, however it may have been too discriminatory, since it was not able to detect relatedness among the *S. hominis* isolates collected in this study. The genetic diversity observed may result from the existence of multiple transposases and integrases in the genome, as revealed by the analysis of the genome available for two *S. hominis* strains. To further clarify the origin of genetic diversity in this species it will be essential to develop a multilocus sequence typing scheme for *S. hominis*.

In spite of the low clonality of *S. hominis*, almost every isolate of this species was found to be multidrug resistant, accumulating resistance to virtually all classes of antimicrobial agents, particularly resistance to aminoglicosides, erythromycin, cotrimoxazole, and fosfomycin. The finding of multidrug resistance among *S. hominis* from this study that are part of an established resident flora may result from the long internment periods and aggressive and continuous antibiotic therapy that neutropenic patients are subjected to. This fact may turn this staphylococcal species into a particularly privileged reservoir and donor for other species sharing the same ecological niche, like *S. epidermidis*, *S. haemolyticus* and *S. aureus* [45].

Another interesting feature of *S. hominis* molecular epidemiology was the finding of a high frequency of SCC*mec* types containing *ccrAB4*, *ccrAB1* and *mec* complex A in its composition, like VI (4B), SCC*mec* VIII (4A) and a new SCC*mec* type with *mec* complex class A associated to *ccrAB1* (1A), and the complete absence of SCC*mec* structures containing *ccrAB2*, *ccrAB3*, *ccrC* and *mec* complex C. Previous studies where SCC*mec* was characterized for only a few *S. hominis* isolates also showed that *S. hominis* predominantly carried, *ccrAB1*, *ccrAB4* and *mec* complex A [18], [46]. Most importantly, we found that *ccrB1* and *ccrB4* from both MRSHo and MSSHo analyzed here had a high homology with those *ccrAB* allotypes of prototype strains of *S. aureus* carrying SCC*mec* type I and VI, respectively. Altogether, the results suggest that *S. hominis*, is an important reservoir of *ccrAB1*, *ccrAB4* and *mec* complex A and could have been the donor of these components to the assembly of SCC*mec* types I, VI, VIII, and the new type 1A. Another observation resulting from our study that sustains this hypothesis was the finding of *ccrB* internal regions of MRSHo (carrying SCC*mec* VI and the new SCC*mec* 1A) with 100% homology with those of MSSHo what suggests that already pre-existing SCC non-*mec* carrying these *ccrAB* allotypes could have been the receptors of *mec* gene complex to give rise to SCC*mec*. Moreover, the finding in our study of a high number of *ccrB1* and *ccrB4* alleles among the small number of *S. hominis* analyzed, suggests that these *ccr* allotypes have been in *S. hominis* long enough to be able to diversify and could have been the species where these *ccr* allotypes were once originated.

In what respects to the origin of SCC*mec* type VI (4B) and VIII (4A), no previous studies documented their link to *S. hominis* or any other species. However, the involvement of *S. hominis* in the assembly of SCC*mec* I was previously suggested due to the finding of a SCC non-*mec* element in MSSHo with high homology with the SCC*mec* I from *S. aureus* [12]. In this study we identified a high number of *S. hominis* isolates carrying a new SCC*mec* type where *ccrAB1* was associated to *mec* complex A, with intact *mecI* and *mecR1*. We believe that this new SCC*mec* type could have been the most direct precursor of SCC*mec* type I that emerged from the latter by *mecI* and *mecR1* IS*1272*-mediated deletion. However, most probably this deletion step did not occur in *S. hominis* since no IS*1272* transposases were found in its genomes (http://www.ncbi.nlm.nih.gov/genome?Db=genome&Cmd=ShowDetailView&TermToSearch=7149;http://www.ncbi.nlm.nih.gov/genome?Db=genome&Cmd=ShowDetailView&TermToSearch=6409).

The methodology used in this study for the definition of SCC*mec* types in MRSHo was only based on PCR data for the two central elements of SCC*mec* – the *mec* complex and *ccr* complex. Although in *S. aureus* this is considered to be a valid approach, for CoNS species the SCC*mec* distribution and structure is still elusive and caution should be taken in interpretation of data. One limitation of the approach used is that it did not take into consideration the proximity of the two elements in the chromosome. Although the conclusions taken are the most parsimonious, the methodology used does not allow us to state that two elements found in the same isolate belong to the same SCC.

**Figure 3:**
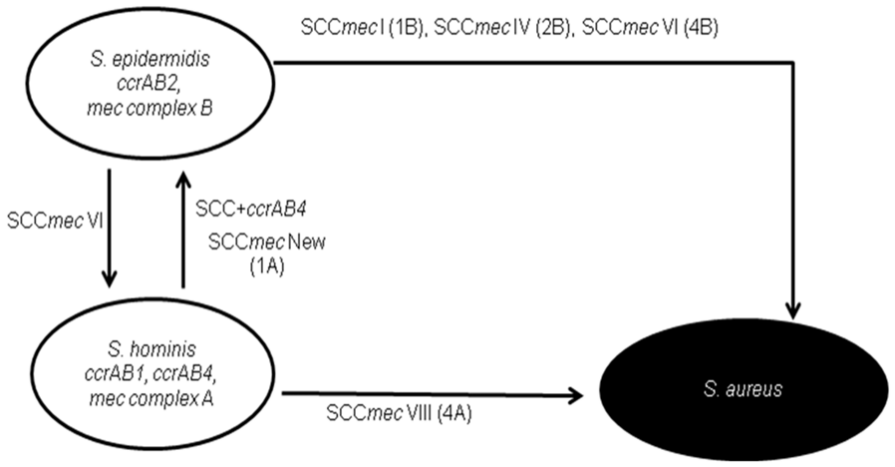
Model for the contribution of *S. hominis* species for SCC*mec* evolution, based on our data and data collected from the bibliography (see discussion). *S. epidermidis* is the primary reservoir of *ccrAB2*, *mec* complex B, and probably was the species where SCC*mec* IV was assembled. *S. hominis* might have been the primary reservoir of *ccrAB1*, *ccrAB4,* and *mec* complex A, and probably was the species where new SCC*mec* type 1A and SCC*mec* VIII were first assembled. SCC*mec* type VI probably resulted from the acquisition by *S. epidermidis* of a SCC non-*mec* carrying *ccrAB4* genes from *S. hominis*. In *S. epidermidis*, this SCC non-*mec* probably received the *mec* complex B that is very frequent in this species and rare in *S. hominis*. According to this model *S. epidermidis* was the most probable donor of SCC*mec* I, IV, and VI, to *S. aureus*, whereas *S. hominis* was the most probable donor of SCC*mec* type VIII to *S. aureus*.

The prevalence of specific *mec* complex classes and *ccr* allotypes observed in this study was previously detected in other CoNS species like *mec* complex C in *S. haemolyticus* and *mec* complex B and *ccrAB2* in *S. epidermidis* [46], [47], suggesting that each CoNS might be a reservoir for specific and different components of the SCC*mec* types identified in *S. aureus* (Figure 3).

The study described here showed that *S. hominis* has a distinctive genetic diversity and has contributed in a specific mode to the assembly of SCC*mec*. The results emphasize the urgent need for studying each CoNS species as a separate entity with its own features and characteristics. Only by understanding the molecular epidemiology of the individual CoNS species it will be possible in the future to design effective infection control strategies against these microorganisms and understand their contribution for the evolution of the broad-spectrum beta-lactam determinant.
